# Treatment outcomes of alcohol use disorder by traditional medicine practitioners using plant derivatives in southwestern Uganda: findings from in-depth interviews

**DOI:** 10.3389/fpsyt.2023.1185108

**Published:** 2023-08-31

**Authors:** Samuel Maling, Jerome Kabakyenga, Charles Muchunguzi, Eunice Apio Olet, Paul Erasmus Alele

**Affiliations:** ^1^Department of Psychiatry, Faculty of Medicine, Mbarara University, Mbarara, Uganda; ^2^Department of Community Health, Mbarara University of Science and Technology, Mbarara, Uganda; ^3^Department of Environment and Livelihood Support Systems, Mbarara University of Science and Technology, Mbarara, Uganda; ^4^Department of Biology, Mbarara University of Science and Technology, Mbarara, Uganda; ^5^Department of Pharmacology and Therapeutics, Mbarara University of Science and Technology, Mbarara, Uganda

**Keywords:** alcohol, traditional medicine practitioners, treatment outcomes, plant derivatives, in-depth interviews, Uganda

## Abstract

**Background:**

Globally, 5.1% of the global burden of disease and injury is attributable to alcohol in addition to its significant negative socio-economic impact. Uganda is ranked among the highest alcohol consuming countries in Africa with a reported alcohol *per capita* consumption of 9.5 liters, much higher than the 6.3 for the African region. Additionally, almost 10% of Ugandans aged 18 and older have an alcohol use disorder. African traditional medicine plays an important role towards universal health coverage in sub-Saharan Africa especially in rural areas. Anecdotal evidence shows that herbal medicines are used by traditional medicine practitioners (TMPs) to treat alcohol drinking problems in Uganda. Data on the outcomes of alcohol treatment by TMPs is scarce. We aimed at documenting the treatment outcomes and secondary benefits of people treated by TMPs using plant derivatives in southwestern Uganda.

**Methods:**

This was a cross-sectional semi-structured qualitative study that investigated alcohol drinking history and treatment outcomes of adults living in Bushenyi district, southwestern Uganda. We used a semi-structured questionnaire to conduct face-to-face in-depths interviews with individuals who had been treated for alcohol drinking problems by TMPs using plant derivatives. Three trained research assistants collected the data using audio recordings backed by field notes. The audio recordings were transcribed verbatim and two independent researchers coded the transcripts guided by *a priori* themes developed by the research team.

**Results:**

We conducted 44 in-depths interviews, majority of the participants (70.5%, 31/44) were male with a mean age of 47 years. Most participants (86.2%, 38/44) consumed spirits in addition to other types of alcohol and the mean duration of alcohol drinking before seeking treatment was 14 years. Most participants (93.3%, 41/44) abstained from drinking after treatment by the TMPs with only 6.7% (3/44) continuing to drink but less amounts. All participants described additional benefits after treatment including improvement in health, family relations and image in society.

**Conclusion:**

People who were treatment for alcohol drinking problems by traditional healers using plant derivatives in this study described personal and social benefits after stopping drinking. This calls for further research to the plants used.

## Introduction

Alcohol is ranked the seventh global contributor of morbidity and mortality accounting for 3.8% female and 12.2% male deaths in those aged 15–49 years (1) in addition to significant socio-economic losses to individuals, families, and communities (2–4). In Africa, alcohol accounts for 6.4% of all deaths and 4.7% of disability adjusted life years (5). Uganda is ranked among the highest alcohol consuming countries in Africa with a reported alcohol *per capita* consumption of 9.5 liters, which is much higher than the 6.3 liters for the African region (6). Besides, 28.8% of adult Ugandans aged 18 years and above are current alcohol drinkers and 9.8% suffer from alcohol use disorders (7). The World Health Organization reports that more than 80% of the African population seek health care from traditional healers, and of those who do so, up to 60% have a diagnosable mental illness (8). Herbal medicine is a major component of African traditional medicine (ATM) and refers to the use of herbal materials, herbal preparations, and finished herbal products intended for human therapeutic use or for other benefits (9). African traditional medicine plays an important role towards universal health coverage among people in sub-Saharan Africa especially in rural areas where 60% of the population reside. ATM is often preferred to modern medicine as it is considered culturally acceptable, affordable and accessible because the traditional medicine practitioners (TMPs) live within local communities. The use of natural plant products for the prevention and treatment of alcohol addiction is described in cultures outside Africa. Wang et al. (10) describe the use of plant materials in reducing voluntary alcohol intake, and improving alcohol drinking behaviors and attenuating withdrawal syndromes of alcohol use disorder. In a systematic review, Lu et al. (11) highlighted evidence that with less cost and perhaps fewer side effects, traditional herbal remedies can complement pharmaceutical treatments for alcohol and other drug withdrawal and relapse prevention. In New Zealand, the role of traditional Maori values, beliefs, and practices in the treatment of alcohol and other substances is acknowledged and considers the sociocultural demands of the Maori people (12, 13).

An estimated 200,000 TMPs exist in Uganda, and 60%–79% of the Ugandan population uses traditional medicine to meet their healthcare needs (14). Traditional healers in Uganda employ a variety of methods for treating mental illness including herbs, appealing to spirits and divine intervention from gods (15, 16). The use of medicinal plants for the treatment of ailments including mental disorders is a common practice in Uganda and other African countries (17–19). Despite alcohol drinking related problems being a major health problem in Uganda, there is limited use of pharmacological therapies in its treatment. Anecdotal evidence shows that herbal medicines are used by TMPs to treat alcohol drinking problems in Uganda. This study aimed at exploring the drinking history, reasons for seeking, treatment outcomes and secondary benefits of people treated by TMPs using plant derivatives in southwestern Uganda.

## Methods

### Study design and setting

This was a cross-sectional semi-structured qualitative study that used in-depth interviews to investigate alcohol drinking history and treatment outcomes of people who were treated by traditional medicine practitioners using plant derivatives. We conducted the study in rural communities of Bushenyi district in southwestern Uganda and data was collected from July to August 2020. Bushenyi district has population of 246,100 people (20) with the majority (73.8%) living in rural areas. The majority of the people (87%) in the district are engaged in subsistence agriculture while others carry out commercial tea, coffee and bananas cultivation. Other economic activities include fishing; stone quarrying, sand and mineral mining, tourism and lumbering. The district covers an area of 841 square kilometers and is home to two natural forests namely Kalinzu and Imaragambo that together cover 84 square kilometers (21). The main tribes are the Banyankole who are the majority, Bakiga, Baganda and the Bakonjo and the main spoken local language is Runyanore-Rukiga. Administratively Bushenyi district is divided into 9 sub-counties, 64 parishes and 565 villages. The district lies 0.4871° S, 30.2051° E, has low-lying plateaus interfaced with hilly terrain and gets 1,500–2,000 mm of rainfall annually. We chose Bushenyi district as the study area because its traditional healers are organized into an association, called the Bushenyi Medical and Traditional Healers Association (BUMETHA) (22) that was founded in 1988 and currently has a membership of over 75 traditional healers. The association promotes collaboration with modern medicine and participates in research some of which has been published (22).

### Study participants

We enrolled individuals who had sought treatment for alcohol drinking problems from traditional medicine practitioners (TMPs) and were treated using plant derivatives.

### Sample size and sampling

Our sample size of 44 participants for the in-depth interviews was guided by a similar previous study in Uganda (23) as well as sample size estimation for qualitative studies as described by Morse (24). We used traditional medicine practitioners who treat alcohol drinking problems using plant derivatives to identify the clients they had treated in the past. The chairperson of the traditional healers recommended the initial contacts and the rest were identified using snowballing. To be included in the study, the client treated had to be reachable for a face-to-face in-depths interview and residing within Bushenyi district.

### Data collection process

Data was collected through face-to-face in-depths interviews using a semi-structured interview guide developed by the research team. The interview guide collected data on socio-demographics of the participants, history of alcohol drinking, reasons for seeking treatment, treatment outcomes and perceived benefits following abstinence. The in-depth interviews were conducted by three research assistants both conversant with the local culture and fluent in the local language (Runyankore-Rukiga) as well as English. The three research assistants had experience of working with traditional healers in research and collaboration projects with traditional medicine practitioners in southwestern Uganda. Additionally, they had a health background, two in mental health and the other in public health. All interviews were conducted in the Runyankore-Rukiga. The interviews were audio recorded and backed by field notes with participant’s consent. Two research assistants collected data each time. One member conducted the interview while the other recorded the interview with an audio recorder and taking notes. Each interview lasted about 60 min. Details of the interview guide is shown in Table 1.

**Table 1:** The in-depth interview guide used to explore drinking history, reasons for seeking and treatment outcomes of people treated by traditional medicine practitioners for drinking problems in southwestern Uganda.

Interview area	Guiding questions
Socio-demographic characteristics	Age, gender, main occupation, other occupations, marital status, education level
Drinking history	Please share the types of alcohol that you drink or were drinking before you went to the traditional healer for treatment? (probe: both locally brewed/home-made and factory manufactured)
Reasons for drinking	Please share what made you to drink alcohol excessively? (probe: perceived beneficial effects, external influence)
Duration of alcohol drinking before seeking treatment	How long had you been drinking alcohol before you went to see the traditional medicine practitioner?
Reasons for seeking treatment	Please share the problems related to you drinking of alcohol that made you visit the traditional medicine practitioner seeking treatment?
Alcohol use disorder treatment outcome	Did you get healed from your drinking after treatment by the traditional medicine practitioner? (probe: stopped completely, continued to drink like before, drunk even more)
Perceived benefits following treatment for alcohol use disorder	Please share the benefits you observe on yourself after you were treated for your drinking problem by the traditional medicine practitioner? (probe further)

Probes are included in brackets where applicable.

### Quality control

The research assistants were trained on study objectives, informed consent and data collection procedures. The in-depth interview guide was pretested among clients of a traditional healer in a suburb of Mbarara city. There were debriefing meetings at the end of each day of data collection to check for completeness of the data. The principal investigator provided oversight during the debriefing and attended some of the interviews to ensure that interviews were conducted properly according to the study protocol.

### Data management and analysis

The audio recordings were transcribed verbatim by the research assistants. The transcripts were validated through reading the transcript while listening to the audio recordings by SM. Two independent researchers coded the transcripts guided by *a priori* themes developed by the research team. The *a priori* themes that guided the analysis were history of alcohol drinking, reasons for seeking treatment, outcomes of treatment and perceived benefits following treatment. Any disagreement between the coders was resolved by discussion and consensus. Data on socio-demographics of the participants, history of alcohol drinking, reasons for seeking treatment and treatment outcomes analyzed using simple descriptive statistics in Microsoft Excel 2019 and summarized in frequency tables.

### Ethical considerations

All participants provided individual written informed consent to participate in the study. The consent form was translated into Runyankore-Rukiga for better comprehension. For the participants who were not formally educated and were unable to read and write, the consent form was read to them verbatim and when they consented, they thumb printed on the consent form. The research was reviewed and approved by the Mbarara University Research Ethics Committee (#39/02–20).

## Results

### Socio-demographic characteristics

We enrolled a total of 44 participants into the study. The mean age was 47 years, with the youngest person treated being 23 years and the oldest 80 years. Majority of the participants (70.5%, 31/44) were male. The details are in Table 2.

**Table 2:** Socio-demographics and treatment outcomes of the patients treated by traditional medicine practitioners using plant derivatives in southwestern Uganda.

Socio-demographic characteristic	Number	%
*Sex*		
Male	31	70.5
Female	13	29.5
*Main occupation*		
Formally employed	16	36.4
Farmer	17	38.6
Business	7	15.9
Retired	1	2.3
Other	3	6.8
*Education*		
No formal education	11	25.0
Primary	16	36.4
Secondary	10	22.7
Tertiary	7	15.9
*Marital status*		
Married	30	68.2
Single	8	18.2
Widowed	6	13.6
*Religious affiliation*		
Catholic	15	34.1
Protestant	21	47.7
Pentecostal	5	11.4
Moslem	2	4.5
Seventh Day Adventist	1	2.3
*Treatment outcomes*		
Stopped alcohol drinking	41	93.2
Continued to drink alcohol	3	6.8

### History of alcohol drinking

#### Types of alcohol drunk by the participants before seeking treatment from the traditional medicine practitioners

We found out that most participants drunk more than one type of alcohol, often mixing locally brewed and factory manufactured alcohol. The commonest type of alcohol consumed was spirits (both factory manufactured and home-made), which was drunk by 86.2% (38/44) of the participants. Other forms of alcohol drunk were tonto and kwete (locally brewed alcoholic beverages) used by 65.8% (27/41) of participant while only a few drunk beers. About half of the participants regularly drunk more than one type of alcohol.

#### Duration of alcohol drinking before seeking treatment

In this study, we found that many of the participants had been drinking alcohol for many years before seeking treatment from the traditional healers. Majority of the participants (84.1%, 37/44) had been drinking for 5 or more years before seeking treatment for alcohol related problems from the TMP. The participants had a mean of 14 years’ duration of alcohol drinking before being treated with the shortest duration of 1 year and the longest 46 years.

### Reasons for seeking treatment

Participants in the in-depth interviews were asked to share the reasons why they sought treatment from the TMPs. They cited several reasons for seeking alcohol treatment, notably being concerns with physical health, fear contracting sexually transmitted diseases such as HIV, loss of jobs and income arising from excessive alcohol drinking. Other reasons mentioned included family problems arising from drinking behavior and being a shame and nuisance in the community.

#### Sickness and accidents related to alcohol

Numerous participants sought treatment from the TMPs following involvement in accidents while drunk, some had repeated hospitalizations, loss of consciousness arising from excessive alcohol intake and suffered physical illnesses related to alcohol. Others were involved in risky sexual behaviors while drunk and feared they would contract HIV. One participant narrated: “*I got an accident and lost teeth while drunk, I was also sleeping on an empty stomach because whenever I would drink I would lose appetite for food, I had lost weight*” R21. Other participant said: “*I had several warnings from my boss at work because of being a drunkard, I was even scared that I could be HIV positive because I would sleep with different women whenever I would get drunk, I almost lost my marriage so I decided to ask my friends to refer to any healer and they referred me to a man who healed me*” R16.

#### Wastage of money and job loss

Many of the participants also reported having sought care from the TMPs due to alcohol related wastage of money, loss of jobs and income following excessive alcohol drinking. One participant had this to say: “…*they stole my money like I told you. But what hurts me most when I recall is the way I lost my job in Mbarara and I left without being paid my salary*” R23.

#### Family problems

Many of the participants decided to see traditional medicine practitioners for treatment due to repeated family problems arising from drinking behavior predominantly violence towards the spouse and children. One participant said: “*I realized that my family was falling apart because I was not caring for my wife and children. My wife was ever complaining and she would have left be by now*” R12.

### Shame in the community

Several participants went to see the TMPs for alcohol treatment after having been feeling they were a shame and nuisance in the community. Some had been spending nights outside in the cold after failing to reach home while drunk, been a nuisance in the community by displaying shamming behavior while drunk, while others thought they were a shaming fellow Muslims who are not expected to drink alcohol. A female participant reported: “…*there is a problem, they do not respect anybody who takes alcohol. And if you are a Moslem and you take alcohol the community see you as a shame because they know that Moslems are not supposed to take alcohol*” R22.

### Alcohol treatment outcomes

#### Abstinence from alcohol following treatment by the TMP

The majority of the participants (93.3%, 41/44) abstained from drinking after treatment by the TMPs using plant derivatives, with only 6.7% (3/44) of those treated continuing to drink but of less amounts. The longest reported duration of abstinence from alcohol after treatment for drinking by a TOM was 46 years while the shortest was 1 year.

### Secondary benefits after alcohol treatment

In addition to abstinence from drinking alcohol, the participants described improvement in their lives. The benefits described by the participants included improvement in health, better family function, improved productivity with savings and improved personal and family image in the community.

#### Improved health

Most participants described improvement in physical health following stopping alcohol drinking. They reported better sleep, looking healthier and younger and no longer suffering from repeated common ailments while three of them reported they had regained their “manhood.”

One participant said: “*I got healthier and stronger to do my work after stopping alcohol, I got more committed to my family, I get good sleep and even now I look younger*” R11.

#### Better family

Most participants interviewed reported improved family function following abstinence from alcohol after being treated by the TMPs. They reported partners and children feeling happy, some were reunited with their spouses whom they had separated with due to problem alcohol drinking, others got married and having a stable family was cited by many participants as a benefit. A participant in an in-depth interview said:

“*My home is now clean because I have the time to make it neat, I take care of my grandchildren and my husband very well. Even I look healthy I never used to eat because all the time I would be thinking of going to look for alcohol*” R17.

#### Better productivity

Majority of the participants reported that they became productive and improved their lives. Some participants cited going back to school, retaining jobs, stopped selling household property, several reported saving money and building better houses for their families.

One participant had this to say when asked about benefits of stopping alcohol drinking following treatment by the healer: “*I built a house, I now have goats, I have two bodabodas (motorcycle taxis) and generally there is a big change*” R13.

#### Improved image in the community

Some of the participants appreciated that their image in the community had been restored following treatment by the TMPS for problem drinking. They reported that they can now be trusted, respected and were presentable as better dressed as this participant said: “*I started saving my money, I built a permanent house, my children started respecting me as their father. My children have all finished school. I also now have respect from the community*” R40.

## Discussion

In this study we conducted face-to-face in-depths interviews to explore alcohol treatment outcomes and secondary benefits of people who were treated for drinking problems by traditional medicine practitioners using plant derivatives in southwestern Uganda. We assessed participant alcohol drinking history, abstinence from alcohol consumption following treatment and the perceived benefits. Most of the participants interviewed reported to have abstained from drinking alcohol following the treatment by the traditional healers. All the participants who stopped drinking alcohol enumerated several personal and social benefits. We found that more males than females sought treatment for their drinking problems. Due to our non-random sampling strategy and small sample size, it’s not feasible to compare this finding with those found in other studies. However, significantly higher alcohol use among men than women has been reported in Uganda and elsewhere (7, 25). For example, at Butabika Mental Hospital in Uganda, males were the majority of the patients treated for alcohol use disorders (26). A similar pattern has also been affirmed by the World Health Organization in that in Africa, females are less often current drinkers than males, and when women drink, they drink less than the men (27, 28). The social, cultural, and religious norms and beliefs help to understand some of the alcohol consumption practices in Uganda. Alcohol use is part of Ugandan cultural, religious and social practices. Drinking alcohol is widespread in Uganda and is accepted among all socioeconomic groups; it is only seen as a problem if a person makes a nuisance of himself or others (29). While drinking is socially acceptable for Ugandan men in bars and other public places, women are typically only allowed to drink at home or in their own neighborhood, with friends or family, including their spouse or sexual partner (25, 29, 30). In this study, for the first time in Uganda to our knowledge, we used face-to-face in-depths interviews to demonstrate favorable treatment outcomes and perceived benefits by people who were treated by traditional healers for drinking problems in southwestern Uganda.

Participants in this study reported drinking a mix of homemade and factory brewed alcohol, with the majority drinking spirits. Most had been drinking for prolonged periods of time before seeking treatment. This pattern of alcohol drinking in Uganda which is dominated by spirits has been observed and reported before. The Uganda Youth Development Link organization in its report of “state of alcohol abuse in Uganda” shows that homemade spirits are the most consumed form of alcohol in Uganda (31). A similar pattern is described in the Ministry of Health in the Uganda Alcohol Status Report—2018 (32).

Reasons for seeking help for alcohol drinking problems described by the participants ranged from suffering from medical problems especially while drunk, social problems in the family and loss of image in the community. Seeking help for drinking problems is reported to be rare in Uganda except in emergencies while experiencing life threatening situation such as acute intoxication and loss of consciousness or after sustaining severe physical injuries (29). Similar reasons were cited by participants in our study. Other findings in agreement with ours have been reported by Zewdu and others in Ethiopia (33). Studies have also found that family, friends and neighbors together constituted the single largest group suggesting care to be sought, this is similar to what was reported by some of our participants (34).

We found that most of the participants in our study had been abstinent from alcohol for long periods following treatment by the traditional healers. Though abstinence is seen as the strongest evidence of recovery from harmful alcohol drinking, our evidence is not strong in this regard due to the small sample size. Publications on alcohol treatment outcomes by traditional medicine practitioners using plant derivatives are scarce. Data available are related to alcohol treatment outcomes following treatment at health facilities or addiction treatment centers. For instance, Kalema and others in Uganda report a very low abstinence rate (21%) after 6 months in people seeking care at alcohol treatment centers in Uganda (35). Kalani and others at Butabika Mental Hospital in Uganda reported a high relapse rate (63%) among patients being treated at the hospitals alcohol and drug unit (26). All these falls below the abstinence rate that we found among people treated by the traditional healers using plant derivatives in this study.

Our participants reported personal and social benefits upon stopping drinking. Most reported improved health, better relation with spouse and children, and saving money earned and improved image in the community. These results are consistent with other studies that show there are additional advantages to quitting drinking besides abstinence. Outcomes such as improved health, social wellbeing, occupation, responsibility for own lives and family, have been described in low- and middle-income settings (30, 36, 37).

The strength of this study is the use of in-depths interviews to document not only stopping drinking as an outcome of alcohol treatment, but also describe reported perceived personal, family and societal benefits following abstaining from alcohol. Our study had three main limitations. We used the traditional healers to identify the people they had treated for alcohol problems using plant derivatives. The healers may have only referred us to the people who they knew had improved following treatment. Besides we relied on self-report by those treated and did not confirm abstinence in the laboratory for additional evidence of abstinence. Finally, our sample size was too small to make meaningful quantitative inferences.

## Conclusion

People who were treatment for alcohol drinking problems by traditional healers using plant derivatives in this study described personal and social benefits after stopping drinking. This calls for further research to the plants used.

## Data availability statement

The raw data supporting the conclusions of this article will be made available by the authors, without undue reservation.

## Ethics statement

The studies involving humans were approved by Mbarara University Research and Ethics Committee. The studies were conducted in accordance with the local legislation and institutional requirements. Written informed consent for participation in this study was provided by the participants’ legal guardians/next of kin.

## Author contributions

SM and PA conceptualized and designed the study. SM, PA, JK, and EO refined the proposal. SM implemented data collection. SM, CM, and PA developed the analysis plan. SM drafted the manuscript while PA, JK, CM, and EO reviewed the manuscript. All authors contributed to the article and approved the submitted version.

## Funding

This research was funded by Mbarara University of Science and Technology using a grant from the African Development Bank.

## Conflict of interest

The authors declare that the research was conducted in the absence of any commercial or financial relationships that could be construed as a potential conflict of interest.

## Publisher’s note

All claims expressed in this article are solely those of the authors and do not necessarily represent those of their affiliated organizations, or those of the publisher, the editors and the reviewers. Any product that may be evaluated in this article, or claim that may be made by its manufacturer, is not guaranteed or endorsed by the publisher.

## Abbreviations

ATM: African traditional medicine
BUMETHA: Bushenyi Medical and Traditional Healers Association
TMP: Traditional medicine practitioner
WHO: World Health Organization
